# The Use of Arabic Vowels to Model the Pathological Effect of Influenza Disease by Wavelets

**DOI:** 10.1155/2019/4198462

**Published:** 2019-12-04

**Authors:** Khaled Daqrouq, Abdel-Rahman Al-Qawasmi, Ahmed Balamesh, Ali S. Alghamdi, Mohamed A. Al-Amoudi

**Affiliations:** ^1^Electrical and Computer Engineering Department, King Abdulaziz University, Jeddah 21589, Saudi Arabia; ^2^Electrical Engineering Department, Majmaah University, Majmaah 11952, Saudi Arabia

## Abstract

Speech parameters may include perturbation measurements, spectral and cepstral modeling, and pathological effects of some diseases, like influenza, that affect the vocal tract. The verification task is a very good process to discriminate between different types of voice disorder. This study investigated the modeling of influenza's pathological effects on the speech signals of the Arabic vowels “A” and “O.” For feature extraction, linear prediction coding (LPC) of discrete wavelet transform (DWT) subsignals denoted by LPCW was used. *k*-Nearest neighbor (KNN) and support vector machine (SVM) classifiers were used for classification. To study the pathological effects of influenza on the vowel “A” and vowel “O,” power spectral density (PSD) and spectrogram were illustrated, where the PSD of “A” and “O” was repressed as a result of the pathological effects. The obtained results showed that the verification parameters achieved for the vowel “A” were better than those for vowel “O” for both KNN and SVM for an average. The receiver operating characteristic curve was used for interpretation. The modeling by the speech utterances as words was also investigated. We can claim that the speech utterances as words could model the influenza disease with a good quality of the verification parameters with slightly less performance than the vowels “A” as speech utterances. A comparison with state-of-the-art method was made. The best results were achieved by the LPCW method.

## 1. Introduction

Digital speech signal processing is an efficient tool for diagnosing voice disorders. There are significant speech parameters described in the literature for characterizing and controlling different types of voice. The speech parameters may include perturbation measurements, spectral and cepstral modeling, noise content measure, and nonlinear behavior. The verification task is very popular in the literature to discriminate between different types of voice disorder [1].

Lifestyles of individuals, like smoking and alcohol drinking, can affect their voice quality [2, 3]. A hoarse voice may result because of prematurity of the mucous membrane, which is considered as one of the most common results of smoking and drinking. However, severe voice disorders can result from excessive smoking or drinking where it would be more appropriate to consider pathological speech [4]. Hypernasality is a very common symptom among cleft lip and palate patients, which is considered as a functional origin disorder. Inappropriate control of the velum generates unusual resonance in the vocal and nasal cavities. As a result, patients with this pathology produce voice with an excess of nasalization.

Patients with Parkinson's disease, neurological origin, are usually identified by an excess of tremor, reduced loudness, monotonicity, hoarseness. Multiple voice disorders, functional, neurological, and laryngeal diseases, were investigated in [1]. Illnesses of the pharynx and larynx could characterize acute infections and inflammation, chronic inflammation, or abnormal growths that are more common among adults. There is a list of disorders such as contact ulcers, vocal cord paralysis, laryngeal papillomas, laryngoceles, and cancer that may change significantly the speech signal patterns.

Cold, flu, and throat infection are common diseases that have a link with laryngitis. A laryngeal voice disorder is mainly characterized by hoarseness, breathy voice, and unusual vibration of the vocal cords because of the presence of polyps and nodules. In [5], the authors used LPC and wavelet transform for modeling influenza and smokers' cases by speech signals of Arabic sentences with relatively good performance.

For voice pathology diagnostics, many research studies have been conducted in the literature [1, 6–9], such as wavelet transform, Gaussian classifier, and Mel-frequency cepstral coefficient (MFCC) [5]. The studies showed a range of accuracy rates falling between 81% and 98% for diverse pathologic cases. The Gaussian mixture model was used for pathological voice recognition of vocal fold disease investigation [8], and a range of accuracy rates between 83% and 98% were recorded. Researchers have investigated a variety of ideas to enhance the performance of the feature extraction methods used. A previous study [9] has tested acoustic parameters using multicorpus optimization with a neural network and support vector machine classifier. An LPC, LPCC, MFCC, and PLP were studied for classification by Palo et al. [10], Selvaraj et al. [11], Pao et al. [12], and Sato and Obuchi [13]. Wavelet transform-based feature extraction methods were suggested in many studies [14–18]. A feature extraction method by Fourier transform was proposed by Wang et al. [19].

For conducting the classification, a suitable database should be prepared where the signals are labeled appropriately to describe the class. In this paper, we consider a recorded dataset to study the pathological disorder of influenza by labeling two Arabic vowels and five Arabic separated words. LPC with DWT is used to model the pathological disorder. The paper is organized as follows: Section 1 has the introduction; Section 2 consists of the method; Section 3 presents the results and discussion followed by the conclusion; and References is the last section.

### 1.1. Recorded Dataset

**Figure 1:**
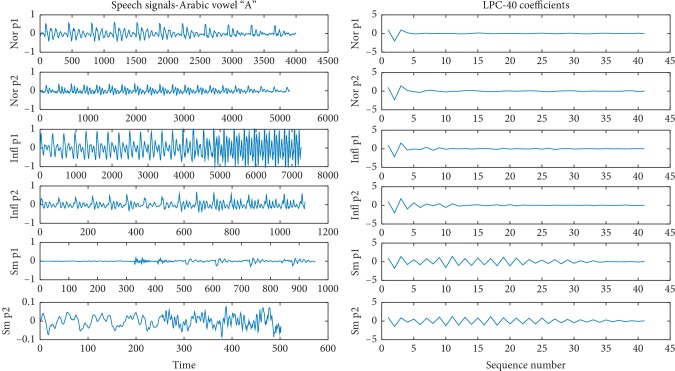
This figure illustrates two normal persons' signals, two influenza persons' signals, and two smokers' signals. For each signal, LPC of forty coefficients are used.

In this study, the Arabic vowels taken from the speech signals are used to model the influenza disease person case by linear prediction coding (LPC) and wavelet transform. To conduct the experiments, 48 male persons (their age from 19 to 23 years) were asked to record their speech signal of the Arabic greeting sentence “alsalam alaykom wa rahmat allahi wa barakato,” that means in English “peace, mercy, and blessings of God,” three times. After that, three “O” and three “A” vowels were chopped out and saved for each person. Sixteen persons that were suffering from influenza and sixteen normal persons were involved in the recording to construct the 48-person database. At the end, we got 144 speech signals of vowels “O” and 144 speech signals of vowel “A.” The speech signals of the sentence were separated into five words to compose 48 separated signals of separated words for influenza cases and the same for normal cases.  Figure 1 illustrates two normal persons' signals, two influenza persons' signals, and two smokers' signals of vowel “A.” For each signal, LPC of forty coefficients is illustrated. The figure shows the ability of modeling the influenza cases where we can easily distinguish the cases by the LPC. Samples of smokers are used in Figure 1 to illustrate the ability of discrimination but are not used in the study. The paper will concentrate on examining and investigating of the possibility of modeling the pathological effect of influenza on the speech signals.

## 2. Method

For modeling the pathological effects of influenza such as hoarseness, breathy voice, and unusual vibration of the vocal cords because of the presence of polyps and nodules, an Arabic vowel speech signal is proposed. The verification process is divided into two main parts:

  First part (the feature extraction part): in this part, DWT subsignals D1, D2, D3, D4, D5, and S5 are calculated by Mallat's algorithm: 
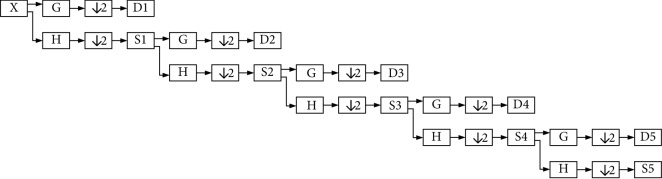
  where D1, in Mallat's algorithm, contains the highest frequency components of the signal and is called the detail DWT subsignal of level *j*. The subsignal D1 was calculated as a convolution of the original signal with the basis *φ*_1,**k**_(**t**) generated from the mother wavelet function and is decimated to get the number of signal samples *N*. For the DWT of level *j* = 1,

(1) $$\begin{array}{c} D1=x\left(t\right)\;*\;\varphi_{1,k}\left(t\right), \end{array}$$

  where,

(2) $$\begin{array}{c} \varphi_{1,k}\left(t\right)=2^{-\;\left(1/2\right)}\varphi \left(2^{-1}t-k\right), \end{array}$$

  and **k** = 1,2,…, **N**/2 and D2 is the second detail DWT subsignal taken from the approximation S1 by convolution and then decimated; so we get S2 also from S1. The same goes for levels 3, 4, and 5 (the last level denoted by *J*). S1, S2, S3, S4, and S5, which is the SJ, are the approximation DWT subsignals of the last level *J* representing the low-frequency part of the original signal *x*(*t*) and are calculated as a convolution of the signal with the basis **φ**_**J**,**k**_(**t**), at each level generated from the father wavelet function [12]:

(3) $$\begin{array}{c} SJ=x\left(t\right)\;*\;\varphi_{J,k}\left(t\right). \end{array}$$

LPC is calculated for each subsignal to compose the feature extraction vector:
  (4) $$\begin{array}{c} FV=\left\{LPC\left(D1\right),\;LPC\left(D2\right),\;LPC\left(D3\right),\;LPC\left(D4\right),\;LPC\left(D5\right),\;LPC\left(S5\right)\;\right\}. \end{array}$$

  This method is denoted by LPCW and was used by the first author in [5].
  Second part: the feature extraction vector is sent to the classifiers KNN and SVM for classification.

## 3. Results and Discussion

**Figure 2:**
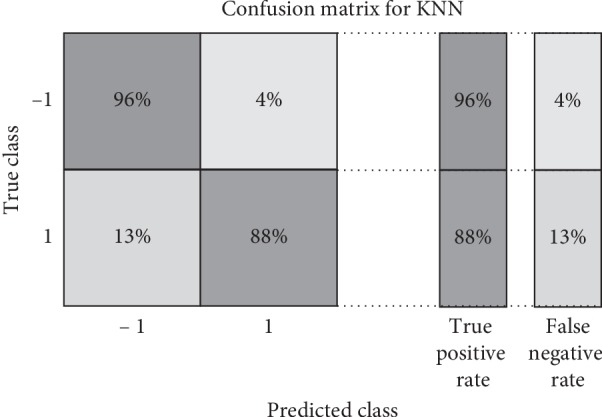
Confusion matrix of KNN classifier illustrating the statistical parameters for the verification task of vowel “A.”

**Figure 3:**
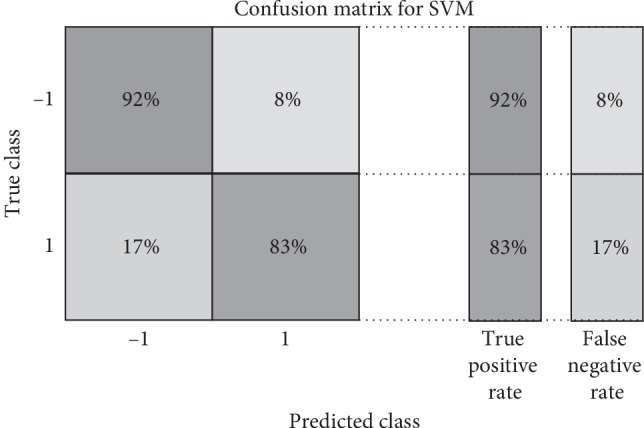
Confusion matrix of SVM classifier illustrating the statistical parameters for the verification task of vowel “A.”

For testing the possibility of modeling the influenza disease by the speech signal of the Arabic vowels on the verification task, many experiments are presented. In the first experiment, the signals of the influenza case with the normal case for “A” and “O” Arabic vowels are tested. The verification task is applied for Influenza/Normal system by LPCW. For classification, *k*-nearest neighbor (KNN) and support vector machine (SVM) are utilized. For KNN with vowel “A,” the parameters were chosen for the best performance as follows: the number of neighbors is 10, the distance metric is cosine, and the distance weight is squared inverse. For SVM, the kernel function is Gaussian, the box constraint is one, and the manual kernel scale is 3.2, which are chosen for the best performance. Three verification or statistical parameters, the true positive rate (TPR), true negative rate (TNR), and accuracy, are calculated for testing, which are calculated as follows:

(5) $$\begin{array}{c} \text{TPR}=\frac{\text{TP}}{P}=1-\text{FNR}, \end{array}$$

(6) $$\begin{array}{c} \text{ACC}=\frac{\text{TP}+\text{TN}}{P+N}, \end{array}$$

where TP is the number of true positives (influenza case correctly identified as influenza), FN is the number of false negatives (influenza case incorrectly identified as normal), *P* is the number of positive cases in the dataset, and *N* is the number of negative cases in the dataset. The results of the verification system for the vowels are tabulated in Table 1. The verification tasks for vowel “O” are also applied for Influenza/Normal system by LPCW, and for classification, *k*-nearest neighbor (KNN) and support vector machine (SVM) are utilized. For KNN with vowel “O,” the parameters were chosen for the best performance as follows: the number of neighbors is 14, the distance metric is a correlation, and the distance weight is squared inverse. For SVM, the kernel function is quadratic, the box constraint is 6, and the manual kernel scale is 3.1, which are chosen for the best performance. The statistical parameters TPR, FNR, and accuracy are also applied. The results are tabulated in Table 1. The obtained results show that the verification parameters achieved for the vowel “A” are better for both KNN and SVM for an average, with an accuracy of 91.7%, and 87.6%, respectively. The results of TPR are also better for the vowel “A” for KNN and SVM for an average, with an accuracy of 96% and 92%, respectively. The classifier KNN shows better performance over the three statistical parameters for the vowel “A” and the SVM for the vowel “O.” A confusion matrix of KNN and SVM classifiers illustrating the statistical parameters for the verification task of the vowel “A” is shown in Figures 2 and 3.

In Table 2, the results of statistical parameters for the verification task of speech utterances as vowels are tested with different cross validations 15 and 25 and holdout-25. The obtained results show that vowel “A” achieves slightly better results for an average. The results for holdout-25 are superior for both classifiers.

In Table 3, the verification system is applied for speech utterances as separated words to be compared with the previous verification system that was applied for speech signals of vowels. The reason behind that is to compare the results of the verification system for the vowels with the results of the verification system for the words and see whether the utterances as words can also model the pathological effect of the influenzas on the speech signals. Ninety-six signals of 48 signals of influenza cases and 48 signals (these signals were also used before to get the truncated vowels) of normal cases are used for testing with cross validation 5. By studying Tables 3 and 4, we can easily claim that the speech utterances as words can model the influenza disease with a good quality of the verification parameters with slightly less performance than the vowels “A” as speech utterances.

**Figure 4:**
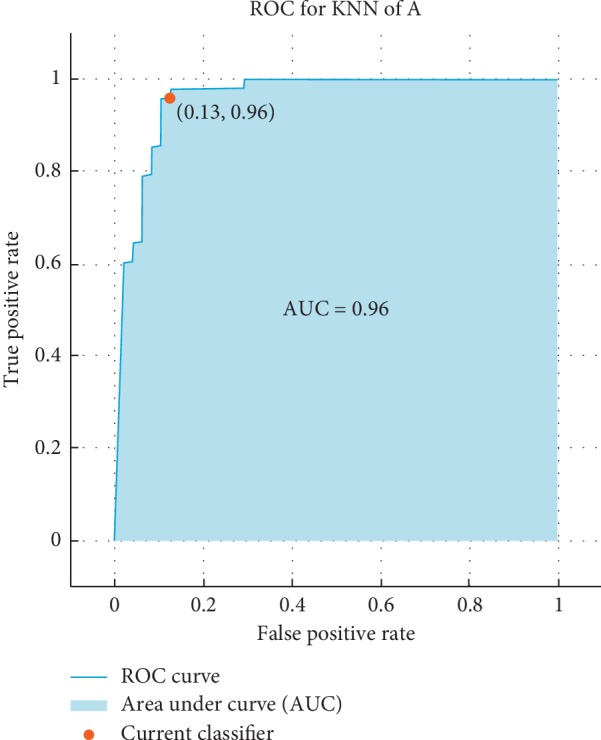
The ROC curve of verification system of vowel “A” for KNN for influenza/normal system.

**Figure 5:**
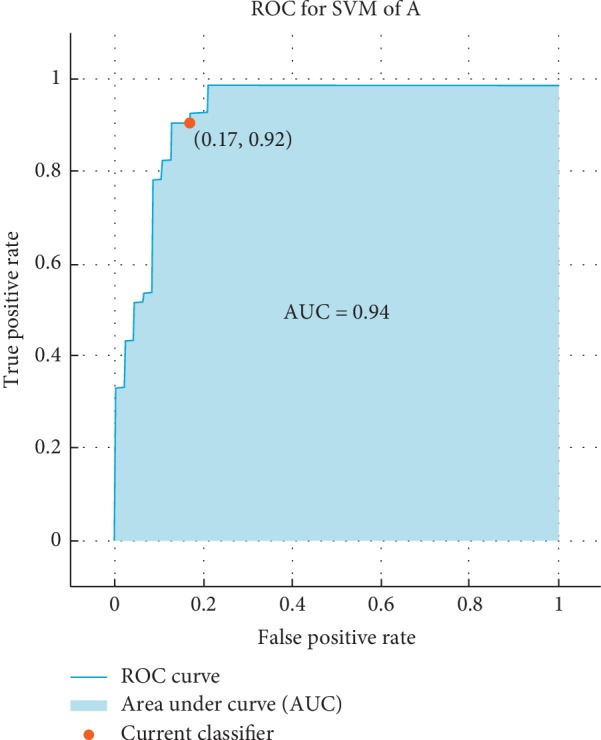
The ROC curve of the verification system of vowel “A” for SVM for influenza/normal system.

**Figure 6:**
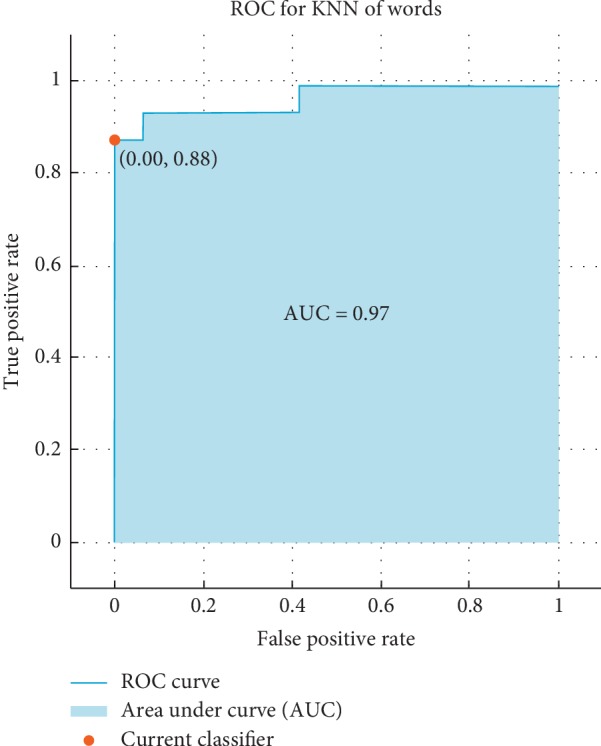
The ROC curve of verification system of words for KNN for influenza/Normal system.

**Figure 7:**
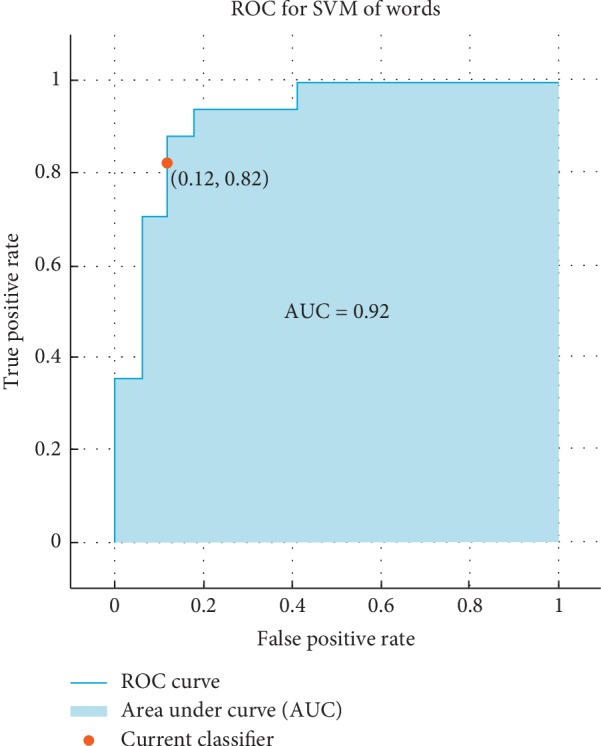
The ROC curve of the verification system of words for SVM for influenza/normal system.

The receiver operating characteristic (ROC) curve, which is a plot of the true positive rate versus the false positive rate for the different possible cutpoints of a verification test, is presented. To claim how much the results are accurate, the ROC is used as follows: the ROC shows that if the result is closer the left-hand border and then the top border of the ROC space it means that it is better. After studying the ROC in Figures 4–7, we can notice that the results for vowel “A” are better than the same of the words based on the area under the curve (AUC) by taking the average of that for KNN and SVM. Additionally, we can claim by observing the ROC curves that the KNN classifier is better than the SVM.

**Figure 8:**
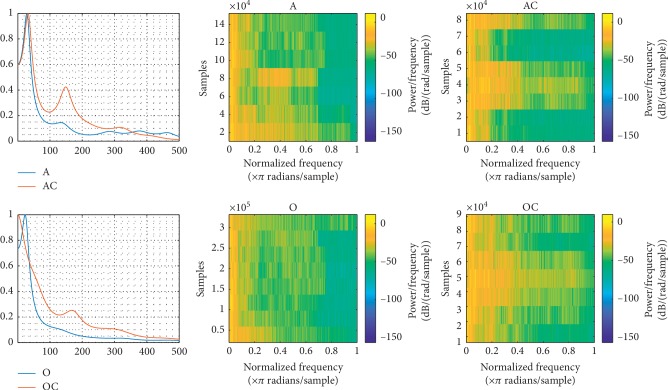
The PSD and spectrogram are illustrated for investigating the possibility of influenza modeling.

To study the pathological effects of influenza on the vowel “A” and vowel “O,” all dataset signals of each vowel are connected in one signal; the power spectral density (PSD) and spectrogram are illustrated for investigating the possibility of the influenza modeling. By observing the PSD of vowels “A” and vowels “A” control (AC) at Figure 8, which is the normal set vowels, we can see how the PSD of “A” is repressed as a result of the pathological effects, where the formants amplitudes of the pathological signals are strongly decreased in comparison with the PSD of “AC.” The same effect can be seen on the PSD of “O” and “OC.” At Figure 8, the spectrogram illustration validates the repression of the formants' frequency amplitudes for influenza by “A” and “O.”

A comparison between different state-of-the-art methods based on the accuracy and efficiency (the average of sensitivity, spesificity, and accuracy) for the verification task of influenza with the speech utterances as vowel “A” as well as separated words with cross validation 5 is investigated in Table 5. Twenty coefficients of LPCC [5], formants [18], and MFCC [10] were involved in the comparison task with LPCW. The results of accuracy for KNN and SVM are calculated for classification parameters of the best performance. The results of all methods show that the vowel “A” is the best for modeling the influenza disease. The best results are achieved by the LPCW method in general, but the formants method is better for the words. The MFCC method shows a good ability to model the influenza disease besides the LPCW method. The method that is based on calculating the LPCC-20 is the worst.

## 4. Conclusions

This study investigates the modeling of the pathological effects on the speech signals of the Arabic vowels “A” and “O.” For feature extraction, LPC and DWT joined with KNN and SVM classifiers for classification has been used. To study the pathological effects of the influenza on the vowel “A” and vowel “O,” all dataset signals of each vowel have been connected in one signal, then the PSD and spectrogram have been illustrated, where the PSD of “A” and “O” was repressed as a result of the pathological effects, because the formants amplitudes of the pathological signals are strongly decreased. The same effect could be seen on the spectrogram illustration that validated the repression of the formants' frequency amplitudes for influenza by “A” and “O.”

The obtained results showed that the verification parameters achieved for the vowel “A” were better for both KNN and SVM for an average. The results TPR are also better for the vowel “A” for KNN and SVM. The KNN classifier has shown better performance over the three statistical parameters for the vowel “A” and the SVM is better for the vowel “O.” The modeling by the speech utterances as words was also investigated. We can claim that the speech utterances as words could model the influenza disease with the good quality of the verification parameters with slightly less performance than the vowels “A” as speech utterances.

A comparison with the-state-of-art methods was made. The best results were achieved by the LPCW method. The formants method was better for the words than vowels. The MFCC method showed a good ability to model the pathology of disease along with the LPCW method. The method that was based on calculating the LPCC-20 was the worst.

## Figures and Tables

**Table 1:** The results of statistical parameters for the verification task of speech utterances as vowels with cross validation 5.

Utter.	Method	System	KNN Acc (%)	KNN TPR (%)	KNN TNR (%)	SVM Acc (%)	SVM TPR (%)	SVM TNR (%)
A	LPCW	Influenza/normal	91.7	96	88	87.6	92	83
O	LPCW	Influenza/normal	85.4	85.4	85.4	88.4	91.7	85.4

**Table 2:** The results of statistical parameters for the verification task of speech utterances as vowels with different cross validations 15 and 25 and holdout-25.

Utter.	Method	System	KNN, CV-15, Acc (%)	KNN, CV-25, Acc (%)	KNN, holdout-25, Acc (%)	SVM, CV-15, Acc (%)	SVM, CV-25, Acc (%)	SVM, holdout-25, Acc (%)
A	LPCW	Influenza/normal	89.7	91.7	95.8	85.4	89.6	95.8
O	LPCW	Influenza/normal	86.5	87.5	97	90.7	90.6	97

**Table 3:** The results of statistical parameters for the verification task of speech utterances as separated words with cross validation 5.

Utter.	Method	System	KNN Acc (%)	KNN TPR (%)	KNN TNR (%)	SVM Acc (%)	SVM TPR (%)	SVM TNR (%)
Words	LPCW	Influenza/normal	94.5	94	94	85.3	82	88

**Table 4:** The results of statistical parameters for the verification task of speech utterances as words with different cross validations 15 and 25 and hangout-25.

Utter.	Method	System	KNN, CV-15, Acc (%)	KNN, CV-25, Acc (%)	KNN, hangout-25, Acc (%)	SVM, CV-15, Acc (%)	SVM, CV-25, Acc (%)	SVM, hangout-25, Acc (%)
Words	LPCW	Influenza/normal	94.5	88.2	97	88.5	82.3	97

**Table 5:** The results of the comparison between different state-of-the-art methods based on accuracy for the verification task of speech utterances as vowel “A” as well as the separated words with cross validation 5.

Utter.	Method	System	KNN, CV-5, Acc (%)	EF	SVM, CV-5, Acc (%)	EF
A	LPCC-20	Influenza/normal	82.5	81.2	75.0	74.7
Words	LPCC-20	Influenza/normal	70.0	70.0	67.0	66.9
A	Formants	Influenza/normal	77.0	75.0	82.3	81.3
Words	Formants	Influenza/normal	81.3	81.2	84.4	84.3
A	MFCC	Influenza/normal	89.6	89.4	88.5	88.5
Words	MFCC	Influenza/normal	85.3	84.4	85.3	84.4
A	LPCW	Influenza/normal	91.7	91.6	87.6	86.9
Words	LPCW	Influenza/normal	94.5	93.2	85.3	82.4

## Data Availability

The data used in the paper can be made available upon request by e-mail address: haleddaq@gmail.com.
